# Coherence and reliability of a wearable inertial measurement unit for measuring postural sway

**DOI:** 10.1186/s13104-019-4238-8

**Published:** 2019-04-02

**Authors:** Eva Ekvall Hansson, Åsa Tornberg

**Affiliations:** 1 Department of Health Sciences/Physiotherapy, Health Science Centre, Lund University, Box 157, 221 00 Lund, Sweden; 2 Department of Health Sciences/Child and Family Health, Lund University, Lund, Sweden

**Keywords:** Balance, Wearable device, Falls

## Abstract

**Objective:**

The aim of this study was to test the coherence of a wearable device, an inertial measurement unit (IMU) against the gold standard; also, to test the intra-trial reliability. This study has a cross-sectional design, where measurement of postural sway in the medio-lateral and anterior–posterior directions was performed simultaneously on a force plate and with a IMU called the Snubblometer (“snubbla” is stumble in Swedish). Thirty-two healthy volunteers participated in the tests.

**Results:**

The coherence between the IMU and the force plate was 0.84 (strong) in the medio-lateral direction with eyes open (EO) and 0.88 (strong) with eyes closed (EC). The ICC for intra-trial reliability for the IMU varied between 0.50 and 0.67 (moderate to good) with a CV between 17.8 and 22.1% and ICC varied between 0.75 and 0.86 (good) for inter-trial reliability, with an SEM of 0.98 to 1.96 mm/s. We have demonstrated that the IMU was both reliable and highly coherent with golden standard, although the two assessment methods were not interchangeable. The ability to move the balance lab out into real life in the form of a wearable device will provide opportunities to perform research that has not been possible before.

**Electronic supplementary material:**

The online version of this article (10.1186/s13104-019-4238-8) contains supplementary material, which is available to authorized users.

## Introduction

Balance is defined as “sensing the position of the body’s centre of mass and moving the body to adjust the position of the centre of mass over the base of support provided by the feet” [1]. Balance can be measured in many different ways and situations, depending on the task and on the objective of measurement; efforts have been made to determine adequate and clinically useful measures of balance for different populations specifically in terms of identifying people at risk of falling [2–4]. However, these efforts do not seem to be sufficient to determine who actually sustains a fall-related fracture [5]. Postural sway is one measure of balance that has been suggested as appropriate for identifying individuals who are at high risk of falling [6]. In particular, the medio-lateral displacement of the centre of pressure in postural sway seems to be able to predict falls [7]. Postural sway is the movement caused by the active feedback control mechanisms responding to the forces of gravity on the body while standing upright [8]. Postural sway is often measured using a force plate [9]; this is suitable for laboratory use but harder to apply in clinical practice. The use of a wearable device as an alternative method of measurement may increase the usefulness of postural sway as measure of balance and of fall risk in clinical practice. However, it is important to first explore the validity of such a device, in terms of coherence and reliability.

The aim of this study was therefore to test the coherence of an inertial measurement unit against the gold standard—a force plate. We also aimed to test the intra-trial reliability of the IMU.

## Main text

### Method

The study is an experimental cross-sectional study.

#### Measurements

The force plate used in the study, representing the gold standard for measuring postural sway (Good Balance™, Metitur Ltd, Finland). The force plates were tested for test–retest and intra-session reliability as well as validity [9–11].

The inertial measurement unit (IMU) used comprises a nine-axis Inertial Measurement Unit (IMU), called the Snubblometer^®^ (‘snubbla’ is ‘stumble’ in Swedish), http://www.Infonomy.com).

#### Participants

Healthy participants were recruited among students and staff at the Health Science Centre, Lund University. Inclusion criteria were: no dizziness or balance problems, no neck pain, no newly acquired injury to the hip, knee, or foot for the last 2 months, and corrected visual impairment, if any. None of the participants had any hearing problems. The group consisted of 32 people: 22 women and 10 men between 20 and 66 years of age (mean 29.8, SD 13.3).

#### Procedure

Postural sway was measured with the subject standing on a force plate while wearing the IMU attached at the lumbar back, in line with L4. Measurements were taken from the force plate and from the IMU simultaneously. The starting position on the force plate was a normal standing position with the arms hanging by the sides and feet in the standardised position with toes facing 30^o^ outwards. The subjects were told to hold the head in a neutral position and to stand as still as possible. Two different measurements were performed for 30 s each, one with the eyes open and another with the eyes closed. The tests were performed three times on each subject, with a resting time of 5 min between them.

#### Statistics

All data were tested for normal distribution. Since the data were normally distributed, parametric statistics was applied. Mean and standard deviations (SD) are used when describing the data. Paired student’s t-test was used when comparing postural sway assessed with the balance plate against the algorithms used to assess postural sway with the IMU. Pearson’s correlation (r_p_) was used for analysis of the coherence between the balance plate and the IMU. A Bland–Altman analysis was performed to detect any systematic variance [12]. Limits of agreement (LOA) and bias were calculated for the Bland–Altman plots to show upper and lower LOA [13]. The p < 0.05 criterion was used for establishing statistical significance.

Test–retest correlations were calculated both for intra-session and inter-session relations. Intra-class correlation coefficient (ICC) and coefficient of variance (CV) were used to analyse intra-session and inter-session reliability. ICC ranges from negative 1 (negative agreement) to 1 (perfect agreement) [14]. All statistical analyses were performed using SPSS for Windows (version 25.0, SPSS Inc., Chicago, Illinois, USA).

#### Ethics

All subjects participated voluntarily and consent was obtained before the tests were carried out. The measures were performed according to guidelines set out by the Helsinki Declaration of 1974. No physical operation was performed and the participants were not influenced by the procedure, either physically or psychologically. The study was approved by the Regional Ethical Review Board in Lund Dnr 2016/585.

### Results

Before the statistical analysis was performed, normal distribution of the data was confirmed. Measures of postural sway on the force plate and with the IMU are displayed in Table 1.

**Table 1:** Postural sway assessed with a force plate (FP) and with an inertial measurement unit (IMU)

	Medio-lateral FP (mm/s)	Medio-lateral IMU (mm/s)	P-value	Anterior–posterior FP (mm/s)	Anterior–posterior IMU (mm/s)	P-value
EC (N = 32)	5.46 (2.56)	6.39 (3.43)	0.001	10.62 (4.44)	13.82 (4.75)	0.001
EO (N = 32)	4.04 (1.61)	5.32 (2.10)	0.001	6.22 (2.37)	8.95 (3.03)	0.001

*EC* eyes closed, *EO* eyes open, *FP* force plate, *IMU* inertial measurement unit

#### Correlation between balance plate and IMU

Analysis showed very high and statistically significant relations between the IMU and the force plate (Table 2).

**Table 2:** Correlations of assessments of postural sway assessed with a force plate (FP) and with an inertial measurement unit (IMU)

	Medio-lateral (FP vs. IMU)	P-value	Bias (mm/s)	LOA (mm/s)	Anterior–posterior (FP vs. IMU)	P-value	Bias (mm/s)	LOA (mm/s)
EC (N = 32)	0.88	0.001	− 0.93	− 4.33 to 2.47	0.81	0.001	− 3.20	− 8.94 to 2.54
EO (N = 32)	0.84	0.001	− 1.29	− 3.59 to 1.01	0.71	0.001	− 2.74	− 7.02 to 1.54

*EC* eyes closed, *EO* eyes open, *FP* force plate, *IMU* inertial measurement unit

#### Bias and limits of agreement

LOA and the values for 2SD is presented in Additional file 1: Figure S1. The Bland–Altman plots for postural sway with eyes closed in the medio-lateral direction show a bias between the assessment on the FP and the IMU of − 0.93 mm/s and LOA − 4.33 to 2.47 mm/s and in the anterior–posterior direction with a bias of − 1.29 mm/s and LOA − 3.59 to 1.01 mm/s. With eyes open, the bias between FP and IMU was − 3.20 mm/s and LOA − 8.94 to 2.54 mm/s in the medio-lateral direction. In the anterior–posterior direction with eyes open, a bias of − 2.74 mm/s was revealed and LOA − 7.02 to 1.54 mm/s.

#### Reliability analysis

No statistical differences were found between the three trials (Table 3) for either of the two methods or with eyes closed or opened. When analysing intra-trial reliability with ICC, the ICC varied between 0.74 and 0.84 for the balance plate and between 0.50 and 0.67 for the IMU. For inter-trial reliability, the ICC spread between 0.89 and 0.94 for the force plate and between 0.75 and 0.86 for the IMU.

**Table 3:** Intra- and inter-trail reliability for the inertial measurement unit and the force plate

	Trial 1	Trial 2	Trial 3	P-value	Intra-trial reliability	CV (95% CI)
	Mean (SD)	Mean (SD)	Mean (SD)		ICC (95%)	
Force plate M-L						
Eyes open (mm/s)	3.88 (1.63)	4.08 (1.45)	4.15 (1.79)	0.80	0.74 (0.58–0.85)	14.7 (11.8–17.6)
Eyes closed (mm/s)	5.85 (2.57)	5.46 (2.97)	5.07 (2.56)	0.48	0.74 (0.58–0.85)	18.4 (15.1–21.7)
Force plate A–P						
Eyes open (mm/s)	6.18 (2.14)	6.28 (2.5)	6.19 (2.54)	0.98	0.82 (0.70–0.90)	11.9 (9.6–14.1)
Eyes closed (mm/s)	11.56 (4.86)	10.29 (4.32)	10.01 (4.09)	0.33	0.84 (0.73–0.91)	15.4 (12.3–18.6)
IMU M-L						
Eyes open (mm/s)	4.96 (2.14)	5.61 (1.99)	5.41 (2.20)	0.45	0.54 (0.34–0.72)	18.9 (14.3–23.4)
Eyes closed (mm/s)	6.85 (3.68)	6.58 (4.15)	5.74 (2.15)	0.41	0.67 (0.50–0.81)	22.1 (17.9–26.6)
IMU A–P						
Eyes open (mm/s)	8.71 (2.51)	9.51 (3.95)	8.65 (2.42)	0.45	0.50 (0.29–0.69)	17.8 (14.3–21.2)
Eyes closed (mm/s)	14.87 (4.99)	13.33 (4.82)	13.27 (4.40)	0.31	0.61 (0.42–0.77)	20.6 (16.9–24.3)

*CV* coefficient of variation, *CI* confidence interval, *ICC* intra-class correlation coefficient, *IMU* inertial measurement unit

### Discussion

This study outlines the coherence and reliability of a wearable device. The main finding of this study was that the IMU displayed strong coherence with measures of postural sway on a force plate, both in the medio-lateral and anterio-posterior directions and both with eyes open and eyes closed. The bias and LOA demonstrated however that the two methods are not interchangeable. The intra-trial reliability for the IMU was high.

#### Coherence

In this study, coherence between 0.71 and 0.88 is to be considered as high. The high coherence found in eyes-closed tests and in the anterior–posterior direction is promising, since this has been difficult to achieve in earlier research [15, 16].

#### Reliability

The ICC in our study ranges from 0.50 to 0.86 and the reliability for the IMU is therefore considered moderate to good. Measures of gait using wearable sensors have been tested, showing good reliability [17] but differences between different models of accelerometers [18].

#### Strengths

A strength of our study was that the assessments were performed in a standardised manner on the same population. The assessments with the IMU and force plate were made simultaneously for the same individual.

#### Implications for future research

A wearable device that can measure postural sway in a valid and reliable way will be beneficial for use both in clinic and in research. The ability to move the balance lab out into real life in the form of a wearable device will provide opportunities to perform research that has not been possible before. However, our findings on healthy people needs to be confirmed on people with different balance disorders.

Our research group is currently performing tests with the IMU attached to the thigh in order to measure gait parameters as well as near falls.

#### Conclusions

The wearable device called the Snubblometer^®^ tested in this study showed a strong coherence with measures of postural sway on a force plate, both in the medio-lateral and anterio-posterior directions and both with eyes open and eyes closed, although the two methods were found not to be interchangeable. The intra-trial reliability for the wearable device was moderate to good and the inter-trial reliability was good.

## Limitations

- The repeated measurements were made at one occasion.
- Making repeated measurement at different day would have made an inter-trail analysis possible.
- The study population in this study is healthy volunteers, who can be assumed have less postural sway than populations with diseases affecting balance.

## Additional file

**Additional file 1: Figure S1.** Postural sway assessed with the force plate (FP) Inertial Measurement Unit (IMU) revealed Bland–Altman plots for average postural sway (mm/s) for Med-Lat sway with (**A**) EC (Bias: − 0.93 mm/s; LOA − 4.33 to 2.47 mm/s) and (**B**) EO (Bias: − 1.29 mm/s; LOA − 3.59 to 1.01 mm/s) and for Ant–Post sway with (**C**) EC (Bias: − 3.20 mm/s; LOA − 8.94 to 2.54 mm/s), (**D**) EO (Bias: − 2.74 mm/s; LOA − 7.02 to 1.54 mm/s).

## Abbreviations

FP: force plate
Med-Lat: medio-lateral displacement
Ant–Post: anterior–posterior displacement
SD: standard deviation
CV: coefficient of variance
r_p_: Pearson’s correlation coefficient
LOA: limits of agreement
ICC: intraclass correlation coefficient
IMU: inertial measurement unit
