# Supplementary material for: Coherence and reliability of a wearable inertial measurement unit for measuring postural sway
Source: BMC Res Notes. 2019 Apr 2;12:201. doi: 10.1186/s13104-019-4238-8 (PMC6446366; doi:10.1186/s13104-019-4238-8)
Supplement: Supplementary file 1 — Additional file 1: Figure S1. Postural sway assessed with the force plate (FP) Inertial Measurement Unit (IMU) revealed Bland–Altman plots for average postural sway (mm/s) for Med-Lat sway with (A) EC (Bias: − 0.93 mm/s; LOA − 4.33 to 2.47 mm/s) and (B) EO (Bias: − 1.29 mm/s; LOA − 3.59 to 1.01 mm/s) and for Ant–Post sway with (C) EC (Bias: − 3.20 mm/s; LOA − 8.94 to 2.54 mm/s), (D) EO (Bias: − 2.74 mm/s; LOA − 7.02 to 1.54 mm/s). [file 13104_2019_4238_MOESM1_ESM.docx]

| A  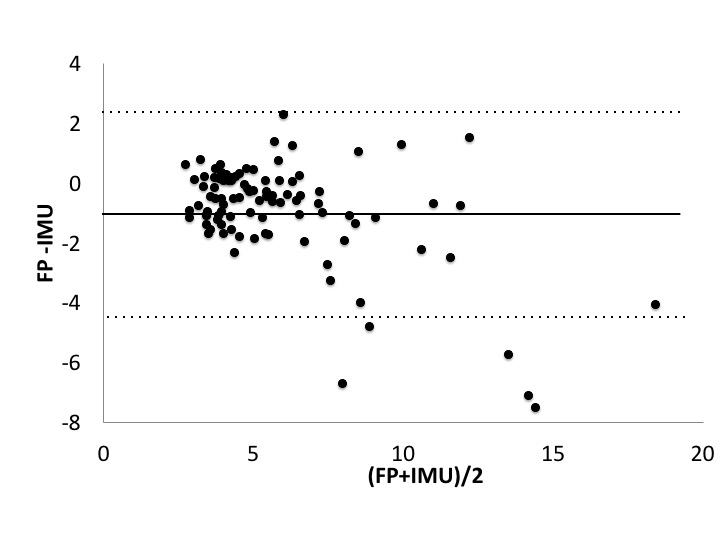 | B  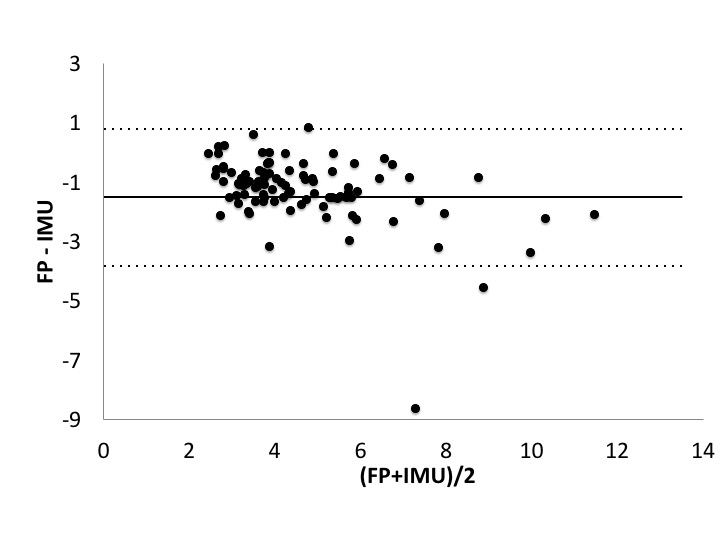 |
| --- | --- |
| C  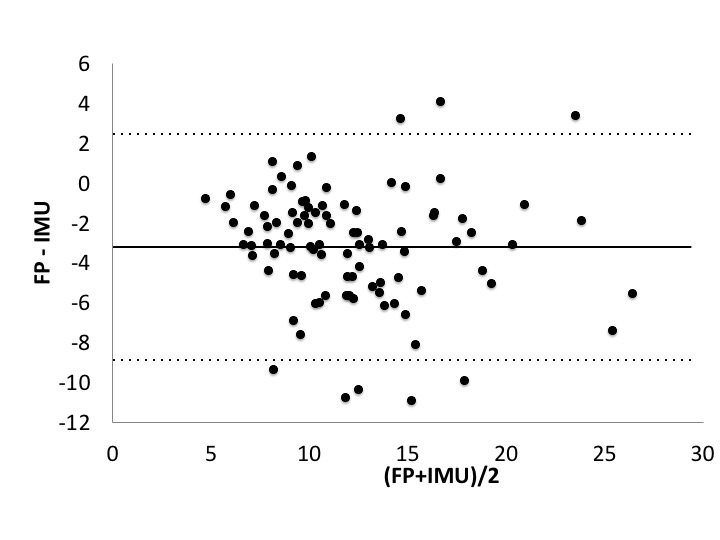 | D  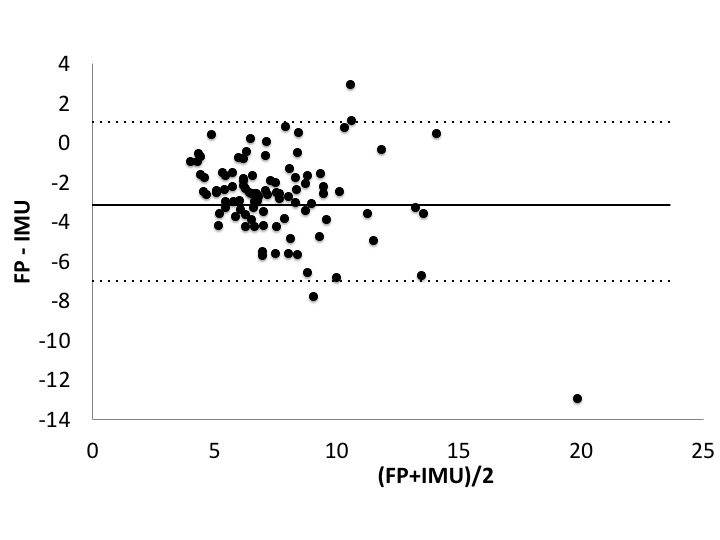 |
| **Figure S1** Postural sway assessed with the force plate (FP) Inertial Measurement Unit (IMU) revealed Bland-Altman plots for average postural sway (mm/s) for Med-Lat sway with (**A**) EC (Bias: -0.93 mm/s; LOA -4.33 to 2.47 mm/s) and (**B**) EO (Bias: -1.29 mm/s; LOA -3.59 to 1.01 mm/s) and for Ant-Post sway with (**C**) EC (Bias: -3.20 mm/s; LOA -8.94 to 2.54 mm/s), (**D**) EO (Bias: -2.74 mm/s; LOA -7.02 to 1.54 mm/s). | |
